# ADDRESSING SOCIAL ISOLATION OF OLDER ADULTS: THE COMMUNITY PERSPECTIVE AND CONTRIBUTION

**DOI:** 10.1093/geroni/igz038.1960

**Published:** 2019-11-08

**Authors:** Bonnie Jeffery, Tom McIntosh, Nuelle Novik

**Affiliations:** University of Regina, Regina, Saskatchewan, Canada

## Abstract

This presentation will discuss the Reducing Isolation of Seniors Collective (RISC), a collaboration of three organizations that have implemented nine projects to address social isolation for rural and urban older adults in Saskatchewan, Canada. A survey was completed with the purpose of identifying community-level awareness, knowledge and perspectives on the extent of social isolation of older adults in their communities. Key variables of interest included contributors to social isolation of older adults, barriers to overcoming social isolation, and community efforts and promising assets for addressing social isolation of older adults in their own communities. To develop an understanding of the extent to which respondents are involved with older adults, the survey asked respondents to report how often they supported, observed, advocated for, and/or interacted with seniors. The 271 respondents identified their roles in the community as human service professionals, healthcare professionals, program facilitators, community leaders, organization members, and community volunteers. Three-quarters of respondents reported that they were involved with seniors at least daily or weekly. While 75.3% believed that social isolation of older adults was “somewhat” or “very” common, almost one-half (41.2%) of respondents believed there was not general awareness of social isolation of older adults by other members in their community. Over one-half of the respondents mentioned community programming (55.7%), friends and neighbours (63.1%), and volunteers (57.2%) as community assets that can reduce isolation of older adults. Respondents reported examples of promising interventions in their communities: church support, library programs, transportation service, visiting programs, advocacy groups, and information sessions.

